# Disablement in the Physically Active Scale Short Form-8: psychometric evaluation

**DOI:** 10.1186/s13102-021-00380-3

**Published:** 2021-12-14

**Authors:** Madeline P. Casanova, Megan C. Nelson, Michael A. Pickering, Lindsay W. Larkins, Karen M. Appleby, Emma J. Grindley, Russell T. Baker

**Affiliations:** 1 University of Idaho, 875 Perimeter Drive, Moscow, ID 83844 USA; 2 Idaho State University, 921 South 8th Ave, Pocatello, ID 83209 USA

**Keywords:** Disablement, Quality of life, Confirmatory factor analysis, Patient reported outcome measures

## Abstract

**Background:**

Patient-centered care and evidence-based practice (EBP) are core competencies for health care professionals. The importance of EBP has led to an increase in research involving clinical outcomes; current recommendations emphasize collecting patient focused measures, thus increasing the need for psychometrically sound patient reported outcome measures (PROMs) of health. Disablement has been identified as a valuable multi-dimensional construct for patient care. The Disablement in the Physically Active Scale Short Form-8 (DPA SF-8) has been proposed as a tool to be used in the physically active population that assesses a physical summary component of health and a quality of life component however, further analysis is necessary to ensure the instrument is psychometrically sound.

**Methods:**

Confirmatory factor analyses (CFAs) were conducted on the DPA SF-8 at each time point to ensure factor structure. Reliability of the scale and internal consistency of the subscales were assessed, and a minimal detectable change (MDC) calculated. Additionally, a minimal clinically important difference (MCID) was also established, and invariance testing across three time points and groups was conducted.

**Results:**

The CFAs at all three visits exceeded recommended model fit indices. The interclass correlation coefficient value (.924) calculated indicated excellent scale reliability and Cronbach’s alpha for subscales PHY and QOL were within recommend values. The MDC value calculated was 5.83 and the MCID for persistent injuries were 2 points and for acute injuries, 3 points. The DPA SF-8 was invariant across time and across subgroups.

**Conclusions:**

The DPA SF-8 met CFA recommendations and criteria for multi-group and longitudinal invariance testing, which indicates the scale may be used to assess for differences between the groups or across time. Our overall analysis indicates the DPA SF-8 is a valid, reliable, and responsive instrument to assess patient improvement in the physically active population.

**Supplementary Information:**

The online version contains supplementary material available at 10.1186/s13102-021-00380-3.

## Background

Health care professionals have an ethical obligation to uphold core competencies, which includes providing patient-centered care and employing evidence-based practice (EBP) [1, 2]. Engaging in EBP involves the integration of the best available research evidence coupled with clinical expertise and unique patient values and circumstances [1, 3, 4]. The need for EBP has led to an increased emphasis on research involving clinical outcomes. Clinical outcomes may be measured using physiological or radiographic findings, patient self-report instruments, or a combination of objective clinical measures and patient-reported outcome measures [5, 6]. The importance of EBP has led to a paradigm shift in measuring clinical outcomes; recommendations have included a reduced reliance on clinician-focused measures (e.g., range of motion scores) and have instead emphasized the need to collect patient focused measures (e.g., the patient’s perspective and experience of their range of motion) [7].

The emphasis on patient focused measures has increased the need for psychometrically sound patient reported outcome measures (PROMs) [3]. The use of PROMs provides a patient-reported assessment of health; PROMs may measure one construct (i.e., unidimensional) or multiple constructs (i.e., multidimensional) and can be categorized as generic (e.g., general health), disease or symptom-specific (e.g., stroke), regional or body-part specific (e.g., shoulder pain), or patient-specific (e.g., occupational performance) [3, 7, 8]. The broad dimensions of health measured by PROMs may include physical function (e.g., mobility, range of movement), symptoms (e.g., pain, fatigue), psychological well-being (e.g., psychological illness, coping), social well-being (e.g., relationships with family, leisure activities), cognitive functions (e.g., concentration, memory), role activities (e.g., employment, financial concerns), personal constructs (life satisfaction, spirituality), satisfaction with care, or a combination of these dimensions [7].

The disablement construct has become an increasingly popular health dimension to assess in patient care. Disablement is a multidimensional construct that combines several dimensions of health status [9]; however, due to theoretical differences in disablement models, various disablement PROMs (e.g., WHO Disablement Assessment Schedule, Duke Health Profile) have been developed for clinical practice. Using PROMs to measure disablement in physically active individuals is particularly important as injury can significantly impair performance in sport and exercise activity. Selecting an appropriate disablement PROM requires consideration of the underlying theoretical model, as well as reflection on the population of interest because researchers have modified or created disablement PROMs to be used in specific subgroups of patients (e.g., physically active patients) and not all are designed for measuring the relevant constructs associated with musculoskeletal injury suffered during sport or exercise [10].

**Fig. 1:**
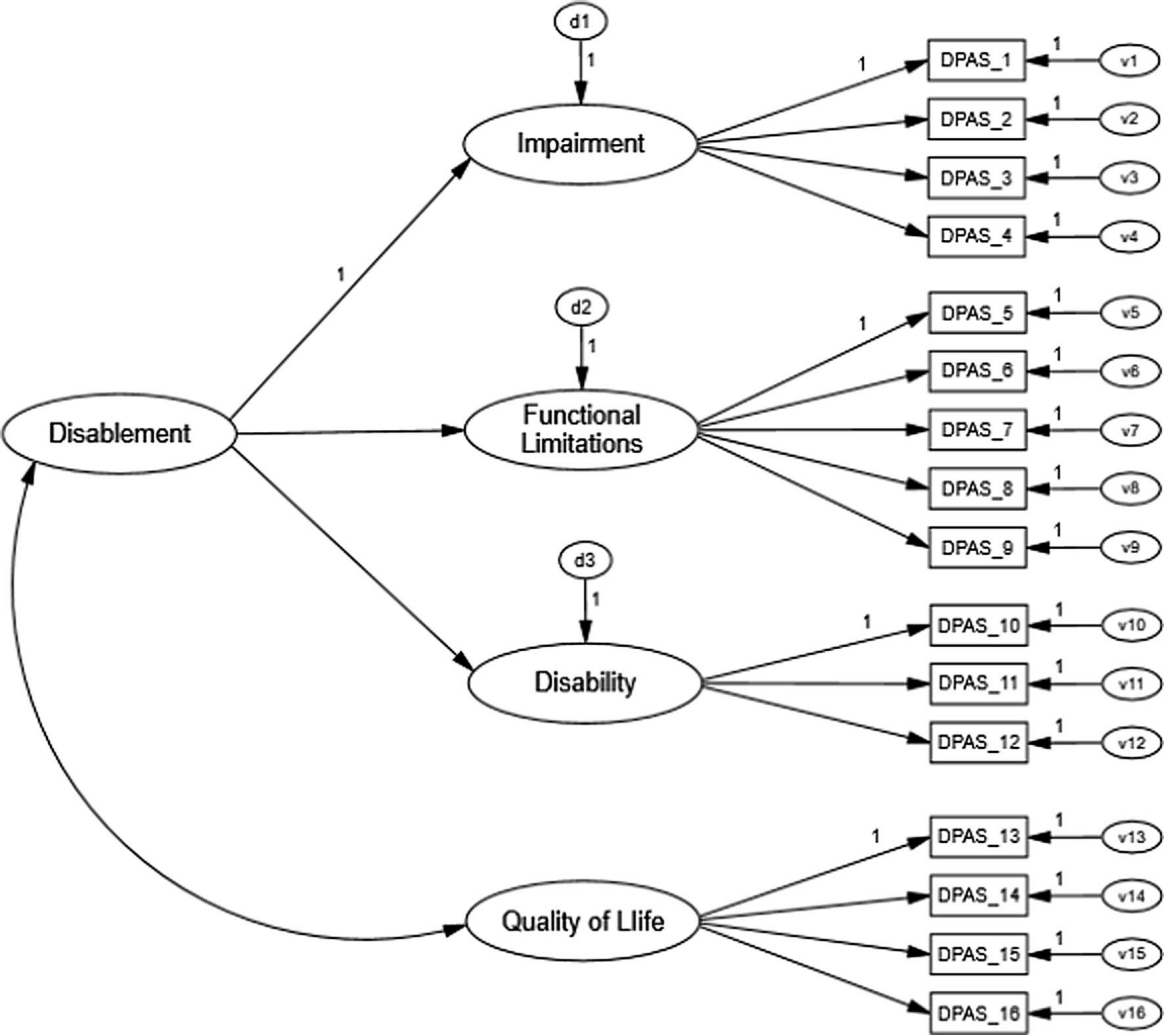
Scale structure of the Disablement in the Physically Active Scale

The Disablement in the Physically Active (DPA) Scale was developed as a multi-dimensional disablement model PROM for a physically active population who suffers musculoskeletal injury while participating in sport and exercise [10, 11]. The DPA Scale is a 16-item scale used to assess transient disablement dimensions of impairment, functional limitation, disability, and quality of life (Fig. 1) [11]. Although the DPA Scale provided clinicians with a much-needed PROM designed for physically active populations (e.g., athletes), subsequent psychometric analysis of the scale indicated the instrument did not meet contemporary recommendations for scale development [12–14]. Specifically, researchers found the DPA Scale did not meet model fit recommendations, had potential issues of multicollinearity between factors, and did not pass testing for invariance across different populations of interests [12, 13]. Alternate model generation using exploratory factor and covariance analysis methods was conducted to resolve the identified issues present in the DPA Scale; a modified, and more parsimonious version of the scale, the Disablement in the Physically Active Scale Short Form-8 (DPA SF-8), was proposed [13].

The DPA SF-8 uses 8-items from the original DPA Scale to assess two factors of disablement: a physical summary component and a quality of life component [12, 13]. The DPA SF-8 exceeded contemporary standards for model fit and accounted for a substantial amount of variance in participants’ scores on the DPA Scale [12, 13]. The modified scale could reduce response burden for participants and provide more efficient administration for clinicians with improved scale validity and precision. Prior to adoption into clinical practice, however, further analysis is necessary to ensure the DPA SF-8 is psychometrically sound (e.g., reliable, valid, responsive) and can accurately assess disablement across subgroups and across time [15, 16].

Thus, further research on the DPA SF-8 is needed to establish scale validity and should include multiple steps to ensure the scale is suitable for use in clinical practice and research: (1) a sample of individuals who only answered the 8-items contained in the DPA SF-8 version must be tested to confirm the factor structure [12, 13], (2) scale reliability and responsiveness must be examined, (3) a minimal clinically important difference (MCID) value should be established to allow clinicians to evaluate if a patient has undergone a clinically significant change, and (4) invariance testing should be conducted across groups and with repeated use of the scale in practice. Therefore, the purpose of the study was to evaluate the psychometric properties of the DPA SF-8 in a three-step process: (1) a confirmatory factor analysis (CFA) of factor structure, using contemporary fit recommendations in a large heterogeneous sample to ensure model fit in respondents who only respond to the items included in the DPA SF-8; (2) psychometric analysis of scale reliability, validity, and responsiveness; and then (3) invariance testing of the scale across subgroups (e.g., sex, age, injury classification) and across visits (e.g., intake, discharge).

## Methods

Athletic training clinics (NCAA Division I = 2; NCAA Division III = 2; High School = 2) and outpatient rehabilitation clinics (large independent clinic = 1; university-based clinic = 1) across the United States were used to recruit participants. Athletic trainers at each site admitting the participants explained the study and corresponding study packet. The study packet included the DPA SF-8, a numeric pain rating scale, a global functioning scale, a patient specific functional scale, the Global Rate of Change Scale (GRoC), and a demographic information questionnaire. All participants provided informed consent and when necessary, legal guardians provided consent prior to participation and minors provided assent. Approval from the university institutional review board was obtained prior to collection of participant information. All data was deidentified prior to being input into Qualtrics for data analysis or being provided to the research team.

### Participants

The targeted sample for recruitment included individuals who were physically active in sport or exercise (Additional file 1: Table S1), while those who were sedentary or inactive were excluded. Participants and the attending athletic trainer were provided the definitions of ‘physically active,’ ‘athlete status,’ and ‘activity level classification;’ clinicians and participants provided a classification for ‘athlete status’ and current ‘activity level’ from the provided options/definitions (Additional file 1: Table S1). Additionally, individuals who were healthy or had an injury classified as acute, subacute, or persistent were recruited while those with chronic pain were excluded from the study (Additional file 1: Table S1) [11, 17]. Participants were grouped by sex, pre-defined athletic status (i.e., competitive athlete, recreational athlete, occupational athlete, physically active in activities of daily living) and injury categories (Additional file 1: Table S1).

### Instrumentation

Participants completed the study packet with the same athletic trainer at three different visits; time of survey packet completion was determined by injury category, consistent with previous research [11]. Healthy individuals or individuals with either an acute or subacute injury, completed the packet at initial intake (visit 1), 3–5 days post initial visit (visit 2), and 7–10 days post initial visit and/or at discharge (visit 3). Individuals with a persistent injury, completed the packet at initial intake (visit 1), 7–10 days post initial visit (visit 2), and 3 weeks post initial visit and/or at discharge (visit 3). Healthy participants completed the packet at the three-time intervals described above. Injured participants completed the initial intake portion of the study packet after suffering an injury but prior to their physical examination at the first visit; injured participants received care from the participating athletic trainer and completed the subsequent sections of the study packet at the time intervals described above based on their injury type as identified by the treating athletic trainer. All de-identified survey data and demographic information were inputted into Qualtrics (Qualtrics, LLC, Provo, UT) by the collecting athletic trainer or by a member of the research team.

#### Disablement in the Physically Active Scale Short Form-8

**Fig. 2:**
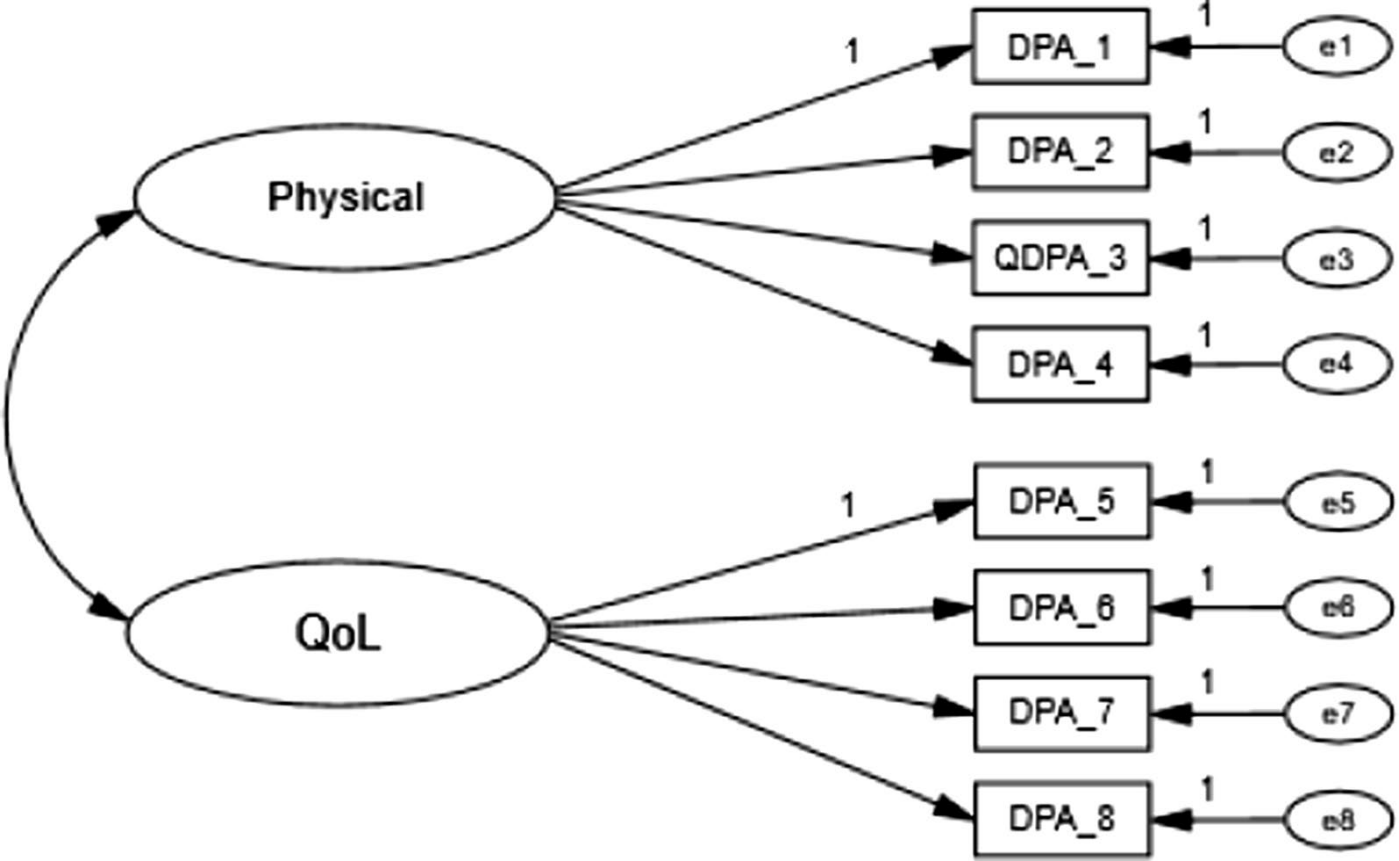
Scale structure of the Disablement in the Physically Active Scale Short Form-8

The DPA SF-8 is an 8-item PROM designed to measure two factors with four items in each latent factor: ‘Physical’ (PHY) and ‘Quality of Life’ (QOL). The two latent factors, PHY and QOL are first order latent variables that covary (Fig. 2). Participants rated each item on a 1 (“no problem”) to 5 (“severe”) Likert scale. The scores provided for each item were then added together, with 8 points being subtracted from the summed total to produce a final total score. Participant total scores could range from 0 to 32 points. The DPA SF-8 was collected at all three visits.

#### Numeric pain rating scale

The Numeric Pain Rating Scale (NPRS) [18] is an instrument designed to measure intensity of pain. Participants were asked to rate their current, best, and worst pain levels over the past 24 h. Participants used a 0 (“no pain”) to 10 (“worst pain imaginable”) scale. A score that represents a patient’s level of pain over 24 h was calculated by averaging the best, current, and worst pain scores reported [19, 20]. The NPRS was collected at all three visits.

#### Global functioning scale

The Global Functioning (GF) scale is a single item question used to assess an individual’s perceived overall level of functioning. Participants used a 10-cm line anchored by 0 (“unable to function at a normal level”) and 100 (“able to function completely at a normal level before the injury/problem”) scale to report overall level of function [21]. The GF scale was collected at all three visits.

#### Patient specific functional scale

The Patient Specific Functional Scale (PSFS) assesses participant’s perceived ability to function on specific activities or tasks. Participants were asked to select three important activities that they are currently not able to do or have difficulty doing as a result of the injury/problem [22–24]. After selecting three activities, participants used a 0 (“unable to perform activity”) to 10 (“able to perform activity at the same level as before injury or problem”) scale to rate each individual activity [22–24]. The PSFS was collected at all three visits.

#### Global rate of change scale

The Global Rate of Change Scale (GRoC) was used to assess an individual’s rate of change throughout treatment. The GRoC has been proposed as the “gold standard” for change and has been previously validated in a number of studies [23, 25–28]. Participants used a 15-point scale (− 7 = a very great deal worse, 0 = unchanged, 7 = a very great deal better). The GRoC was collected at the second and third visits.

#### Demographic information questionnaire

The de-identified participant demographic information collected at the first visit included injury category (i.e., persistent, acute, sub-acute, or healthy), patient athletic status (e.g., competitive athlete, recreational athlete), age, sex, sport, general injury location (e.g., lower extremity, upper extremity), specific injury location (e.g., head/neck, shoulder/arm, ankle/foot), and type of injury (e.g., arthritis, sprain, post-surgery).

### Data analysis

All data was input into Qualtrics (Provo, UT) by the athletic trainer or a member of the research team. Data were then downloaded and analyzed using Statistical Package for the Social Sciences (SPSS Inc., Chicago, IL, USA) Version 26 and Analysis of Moment Structure (AMOS, SPSS, Inc.) Version 26. Missing responses were calculated for the DPA SF-8 and individuals who were missing more than 10% of the items were removed from the data set. Although demographic data was assessed, individuals missing information were not removed from the data set. After missing data was calculated, assessment of univariate and multivariate outliers was conducted. Data normality was assessed by examining histograms, skewness values, kurtosis values, and examining for outliers using z-scores and Mahalanobis distance. Participants with z-scores exceeding |3.4| for an individual item were flagged and removed. Multivariate outliers for each individual were assessed, flagged, and removed from the data set if the Mahalanobis distance exceeded the cut-off value identified in the chi-square table with degrees of freedom (*p* = 0.01) [29]. For longitudinal invariance, individuals who did not respond to DPA SF-8 items at all three time points (i.e., visits) were not used in the analysis.

#### Scale structure

The full sample was used to conduct confirmatory factor analyses (CFA) with maximum likelihood estimation in Analysis of Moment Structures (AMOS) software (IBM Corp., Armonk, NY) on the proposed 8-item, 2-factor structure of the DPA SF-8 by time point (i.e., visit; Fig. 2). Model fit indices were evaluated based on a priori values. The relative goodness-of-fit indices computed were the Comparative Fit Index (CFI; ≥ 0.95), Tucker-Lewis Index (TLI; ≥ 0.95), Root Mean Square Error of Approximation (RMSEA ≤ 0.06), and Bollen's Incremental Fit Index (IFI; ≥ 0.95). Additionally, the likelihood ratio statistic (CMIN) was assessed but not used as the primary assessment measure because it is heavily influenced by sample size [15, 29]. Because model fit criteria were met, longitudinal and multigroup invariance testing was conducted.

#### Reliability

Internal consistency of the scale was assessed by calculating Cronbach’s alpha for each proposed factor; Cronbach’s alpha was set a priori at ≥ 0.70 and ≤ 0.89 [30, 31]. Additionally, three intraclass correlation coefficients (ICC; 2,1) were calculated to assess test–retest reliability for the DPA SF-8 total scores, PHY subscale scores, and QOL subscale scores for healthy individuals across time points (i.e., visits). Values were set a priori: < 0.50 = poor, 0.50–0.75 = moderate, 0.75–0.90 = good, > 0.90 = excellent [32]. The standard error of measurement (SEM) value was then computed for total scores, PHY subscale scores, and QOL subscale scores using the formula SEM = SD × √1 − ICC. Minimal detectable change (MDC) values represent the smallest change that does not result from measurement error and three MDC values were calculated with the formula MDC = SEM × 1.96 × √2 for each ICC value [33].

#### Validity

Correlations were assessed using a covariance modeling approach between the second-order latent variable of the DPA SF-8 and the scores of the GF, NPRS, and PSFS. Additionally, correlations were assessed between the first-order latent variables of the DPA SF-8 (i.e., PHY, QOL) and the GF, NPRS, and PSFS at each time point (i.e., visit).

#### Responsiveness

Responsiveness is typically understood as an aspect of validity for longitudinal research; it is the ability of an instrument to detect change over time [34, 35]. Clinical instruments used in an evaluative manner (e.g., is my patient getting better throughout treatment) should adequately detect changes related to the measure of interest. To detect responsiveness, a protocol from previous research establishing the responsiveness of the original DPA Scale was used [11]. The protocol included creating four Receiver Operating Characteristics (ROC) curves for the participants in the study who were classified as injured and received care from their athletic trainer: two for individuals with acute or subacute injuries and two for individuals with persistent injuries. The procedure involved creating change scores for the DPA SF-8 and for classification group scales (i.e., NPRS, GF, PSFS). First, two change scores from the DPA SF-8 were calculated by subtracting the scores from visit 2 with visit 1 (V2 score) and subtracting scores from visit 3 with scores from visit 2 (V3 score). Then change scores were calculated for the NPRS, GF, and PSFS scales by subtracting the scores from visit 2 with visit 1 and subtracting scores from visit 3 with scores from visit 2.

The change scores from the DPA SF-8 (i.e., V1, V2) were then used to calculate the plots for the ROC curve based on classification groups (i.e., clinical significance, stable) that would indicate undergoing a clinically significant change. Due to the multi-dimensional nature of the DPA SF-8, as well as potential limitations of the GRoC for assessing change [36], change scores from four different scales were used as criteria for determining clinically significant changes across treatment. Scores from the GRoC and the change scores from the NPRS, GF, and PSFS were used as criteria for classification groupings; individuals were placed into two classification groups: one group for visit 2 and one group for visit 3. To be placed in the clinically significant group an individual had to meet two criteria: GRoC score of 4 or greater [10, 25], NPRS change score difference of 30% or more [22], PSFS change score of 2 or greater [22, 37], or GF change score difference of 30% or more; criteria were determined by selecting validated MCID values for each scale. If an individual did not meet at least two of the four criteria, the participant was placed in the stable group.

Sensitivity and specificity values were then calculated for V2 and V3 based on the number of individuals classified as experiencing a clinically significant change versus those who did not experience a clinically significant change (i.e., stable). A ROC curve was plotted using the sensitivity and specificity values. The area under the curve (AUC) was used to determine if the DPA SF-8 would correctly distinguish between individuals with a clinically significant change and those who did not experience a clinically significant change; an AUC value equal to 1.00 indicates the test has perfect discernment between groups [38, 39].

Two ROC curves were calculated using participants with acute or subacute injuries only and two ROC curves were calculated using individuals with persistent injuries only. The MCID value was determined by selecting the point on the ROC curve that represents the smallest overall error rate [40, 41]. The MCID represents the smallest change in score on the DPA SF-8 that a participant would perceive as beneficial and would indicate the patient has experienced a clinically significant change in the variable being measured [11, 40].

#### Invariance testing

The same criteria utilized for the CFAs were used to assess fit for invariance models [15, 29]. Invariance testing with the full sample was conducted to assess measurement invariance of the DPA SF-8 across three visits (i.e., longitudinal invariance) and between subgroups of the sample (i.e., multigroup invariance.). Individuals who completed the DPA SF-8 at all three visits and had suffered an injury were used to assess invariance across time; data from visit one was used to assess multi-group invariance between injury status, sex, and activity levels. Invariance was evaluated based on a CFI difference (CFI_DIFF_) of less than 0.01, and the chi-square difference test (χ^2^_DIFF_), with a *p*-value cut-off of 0.01 [15, 29]. The CFI_DIFF_ test held greater weight in decisions regarding invariance testing model fit because of the sensitivity of the χ^2^_DIFF_ test regarding sample size [15, 29]. Therefore, if a model exceeded the χ^2^_DIFF_ test but passed the CFI_diff_ test, invariance testing would continue.

## Results

A total of 525 individual responses were collected. Of the 525, twenty individuals were missing more than 10% of the responses and were removed from the dataset. Five individuals reported scores that were identified as univariate outliers and 22 reported scores that were identified as multivariate outliers; the 27 individuals were subsequently removed from the dataset. A total of 478 individuals were retained for analysis. The mean age of the sample was 27.52 ± 11.55 years (range = 13–70; median = 22) with males accounting for 47.6% (n = 216) and females accounting for 49.4% (n = 236). Individuals with persistent injuries accounted for 36.2% (n = 177) of the sample and recreational athletes accounted for 33.3% (n = 159) of the sample. The largest group of participants were recreational athletes with medium activity levels. Individuals who met the definitions of ‘physically active’ and ‘physically active in activities of daily living’ were retained because these individuals were not sedentary/inactive; a distinction in classification (i.e., ‘extremely low’ vs. ‘low’) was made because this group did not participate in activity to meet an ‘athlete’ definition for competition, recreation, or occupation (Additional file 1: Table S1). Full demographic information is presented in Table 1.

**Table 1:** Demographic information

	Full sample (n = 478)
**Sex**	N, %
Male	216 (45.2)
Female	236 (49.4)
Other	2 (0.4)
Unknown	24 (5.0)
**Activity level**	
Extremely low	25 (5.2)
Low	105 (22.0)
Medium	179 (37.4)
High	133 (27.8)
Unknown	36 (7.5)
**Athlete Status**	
Competitive Athlete	48 (10.0)
Recreational Athlete	159 (33.3)
Occupational Athlete	25 (5.2)
Activities of Daily Living	126 (26.4)
Unknown	120 (25.1)
**Injury Category**	
Persistent Injury	177 (37.0)
Acute Injury	69 (14.4)
Sub-Acute Injury	89 (18.6)
Healthy	30 (6.3)
Unknown	113 (23.6)
**Clinic Setting**	
NCAA Division I	27 (5.6)
NCAA Division III	39 (8.2)
High School	24 (5.0)
University-based Outpatient Clinic	368 (77.0)
Large Independent Clinic	20 (4.2)
**Ethnicity**	
Caucasian/White	380 (79.5)
African American	7 (1.5)
Hispanic	27 (5.6)
Asian/Pacific Islander	25 (5.2)
Other	14 (2.9)
Unknown	25 (5.2)

### Scale structure

**Fig. 3:**
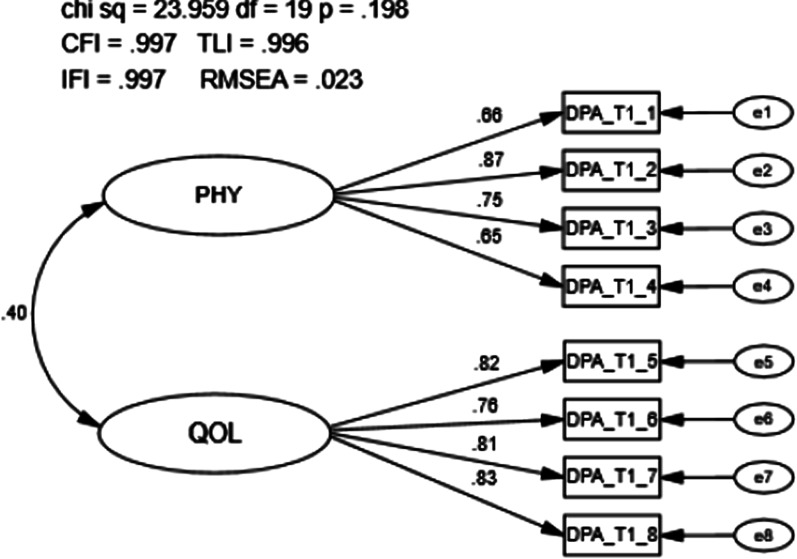
Confirmatory factor analysis of the Disablement in the Physically Active Scale Short Form-8 visit 1. Chi Sq = Chi Square (χ2), CMIN/DF = the χ2 / degrees of freedom ratio; CFI = Comparative Fit Index; TLI = Tucker-Lewis Index; IFI = Bollen’s Incremental Fit Index; RMSEA = Root Mean Square Error of Approximation, df = degrees of freedom, p = alpha level

The scale structure of the DPA SF-8 was assessed at all three time points (Visit 1, Visit 2, Visit 3). Group means are presented in Table 2 by visit and injury type. A total of 478 individuals completed the DPA SF-8 at time 1 (i.e., visit 1) and were used for the analysis. The goodness of fit model indices exceeded recommended values (CFI = 0.997, TLI = 0.996, IFI = 0.997, RMSEA = 0.023; Fig. 3). The first-order latent variable correlation (r = 0.40, r_s_ = 0.16) and factor loadings were significant (*p* < 0.001), with loadings ranging from 0.66 to 0.87. Modification indices did not demonstrate any significant cross-loadings and no meaningful modifications were necessary.

**Table 2:** Group Mean Scores of the DPA SF-8 by Visit and Injury Classification

DPA SF-8 collection visit	Injury category	N	Mean ± SD
Visit 1	Persistent injury	177	12.69 ± 5.46
	Acute injury	69	11.99 ± 5.39
	Sub-acute injury	89	10.75 ± 4.60
	Healthy	30	3.07 ± 4.11
	Total	365	11.3 ± 5.74
Visit 2	Persistent injury	135	9.87 ± 5.71
	Acute injury	52	7.25 ± 5.09
	Sub-acute injury	66	8.17 ± 4.70
	Healthy	29	1.03 ± 2.04
	Total	282	8.08 ± 5.71
Visit 3	Persistent injury	104	8.00 ± 6.26
	Acute injury	35	5.83 ± 4.89
	Sub-acute injury	47	6.98 ± 4.94
	Healthy	28	1.12 ± 2.04
	Total	214	6.52 ± 5.8

**Fig. 4:**
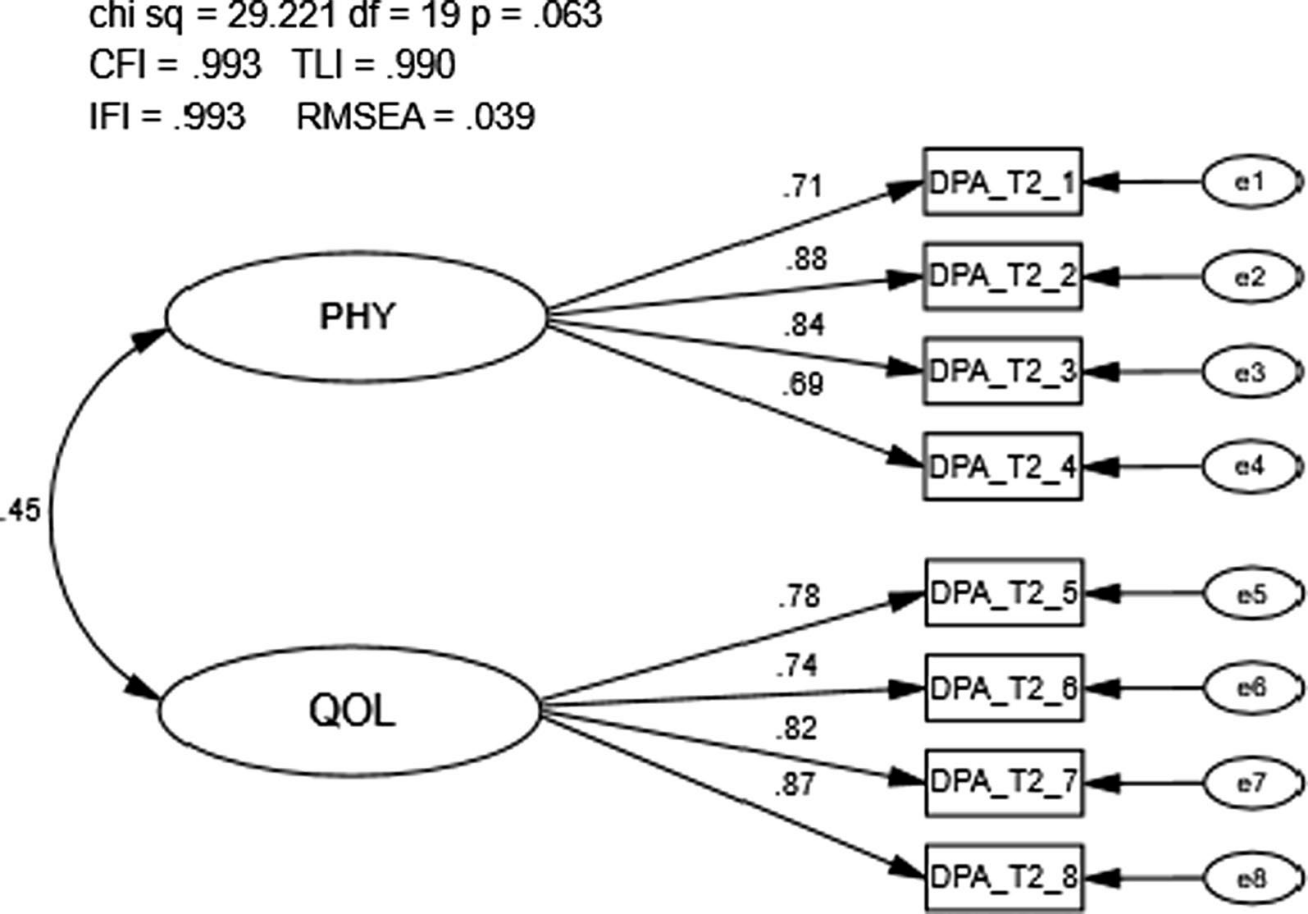
Confirmatory factor analysis of the Disablement in the Physically Active Scale Short Form-8 visit 2. Chi Sq = Chi Square (χ2), CMIN/DF = the χ2/degrees of freedom ratio; CFI = Comparative Fit Index; TLI = Tucker-Lewis Index; IFI = Bollen’s Incremental Fit Index; RMSEA = Root Mean Square Error of Approximation, df = degrees of freedom, p = alpha level

A total of 347 individuals completed the DPA SF-8 at time 2 (i.e., visit 2) and were used for the analysis. The goodness of fit model indices exceeded recommended values (CFI = 0.993, TLI = 0.990, IFI = 0.993, RMSEA = 0.039; Fig. 4). The first-order latent variable correlation (r = 0.45; r_s_ = 0.21) and factor loadings were significant (*p* < 0.001), with loadings ranging from 0.69—0.88. Modification indices did not demonstrate any significant cross-loadings and no meaningful modifications were necessary.

**Fig. 5:**
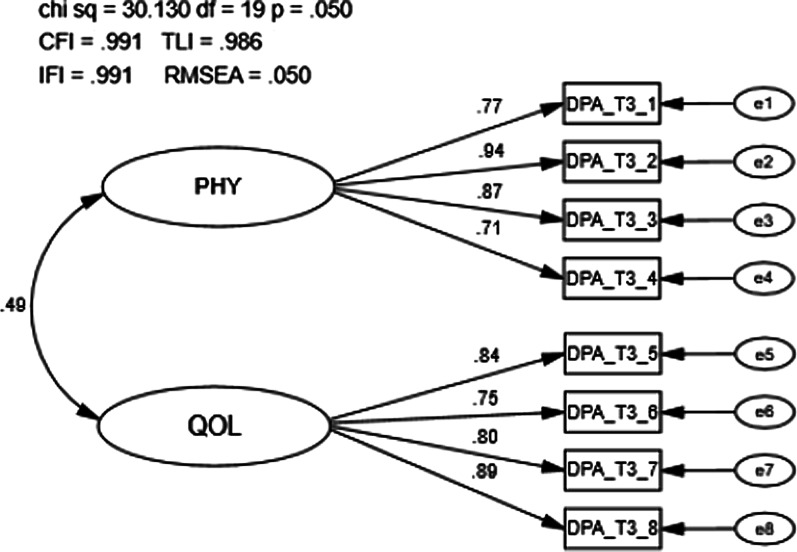
Confirmatory factor analysis of the Disablement in the Physically Active Scale Short Form-8 visit 3. Chi Sq = Chi Square (χ2), CMIN/DF = the χ2 / degrees of freedom ratio; CFI = Comparative Fit Index; TLI = Tucker-Lewis Index; IFI = Bollen’s Incremental Fit Index; RMSEA = Root Mean Square Error of Approximation, df = degrees of freedom, p = alpha level

A total of 234 individuals completed the DPA SF-8 at time 3 (i.e., visit 3) and were used for the analysis. The goodness of fit model indices exceeded recommended values (CFI = 0.991, TLI = 0.986, IFI = 0.991, RMSEA = 0.050; Fig. 5). The first-order latent variable correlation (r = 0.49; r_s_ = 0.24) and factor loadings were significant (*p* < 0.001), with loadings ranging from 0.71 to 0.94. Modification indices did not demonstrate any significant cross-loadings and no meaningful modifications were necessary.

### Reliability

Factor 1, Physical (PHY), included DPA SF-8 items 1–4, and Factor 2, Quality of Life (QOL), included items 5–8. Cronbach’s alpha was assessed by factor (PHY, QOL) across three time points (Visit 1, Visit 2, Visit 3). The PHY factor alphas were 0.81 (Visit 1), 0.86 (Visit 2), and 0.89 (Visit 3) while the QOL factor alphas were 0.87 (Visit 1, 2) and 0.88 (Visit 3). The ICC (2,1) for healthy individuals (n = 26) across visits was 0.924 with an SEM value of 2.10 and an MDC value of 5.83 points. The ICC (2, 1) for the PHY subscale was 0.899 with an SEM value of 1.44 and an MDC value of 4.0. The ICC (2, 1) for the QOL subscale was 0.841 with an SEM value of 1.69 and MDC value of 4.68.

### Criterion (concurrent) validity

The correlations between the second-order latent variable DPA SF-8 and the GF scores were significant at − 0.63 (r_s_ = 0.40, *p* < 0.001) for visit 1, − 0.56 (r_s_ = 0.32, *p* < 0.001) for visit 2, and − 0.65 (r_s_ = 0.42, *p* < 0.001) for visit 3. The correlations between the second-order latent variable DPA SF-8 and the average NPRS scores were significant for visit 1 at 0.58 (r_s_ = 0.34, *p* < 0.001), 0.80 (r_s_ = 0.64, *p* < 0.001) for time 2, and 0.78 (r_s_ = 0.61, *p* < 0.001) for visit 3. The correlations between the second-order latent variable DPA SF-8 and the average PSFS score were significant for visit 1 at − 0.51 (r_s_ = 0.26, *p* < 0.001), − 0.69 (r_s_ = 0.48, *p* < 0.001) for visit 2, and − 0.65 (r_s_ = 0.42, *p* < 0.001) for visit 3.

For visit one, the correlations were significant between the first-order latent variable PHY and the GF score (r = − 0.55, r_s_ = 0.30, *p* < 0.001), the NPRS (r = 0.57, r_s_ = 0.32, *p* < 0.001), and the PSFS (r = − 0.51, r_s_ = 0.26, *p* < 0.001); the correlations were also significant between the first-order latent variable QOL and the GF score (r = − 0.29, r_s_ = 0.08, *p* < 0.001), the NPRS (r = 0.23, r_s_ = 0.05, *p* < 0.001), and the PSFS (r = − 0.21, r_s_ = 0.04, *p* < 0.001).

For visit two, the correlations were significant between the first-order latent variable PHY and the GF score (r = − 0.58, r_s_ = 0.34, *p* < 0.001), the NPRS (r = 0.66, r_s_ = 0.44, *p* < 0.001), and the PSFS (r = − 0.64, r_s_ = 0.41, *p* < 0.001); the correlations were also significant between the first-order latent variable QOL and the GF score (r = − 0.24, r_s_ = 0.06, *p* < 0.001), the NPRS (r = 0.44, r_s_ = 0.19, *p* < 0.001), and the PSFS (r = − 0.34, r_s_ = 0.12, *p* < 0.001).

For visit three, the correlations were significant between the first-order latent variable PHY and the GF score (r = − 0.64, r_s_ = 0.41, *p* < 0.001), the NPRS (r = 0.72, r_s_ = 0.52, *p* < 0.001), and the PSFS (r = − 0.64, r_s_ = 0.41, *p* < 0.001); the correlations were also significant between the first-order latent variable QOL and the GF score (r = − 0.33, r_s_ = 0.11, *p* < 0.001), the NPRS (r = 0.43, r_s_ = 0.18, *p* < 0.001), and the PSFS (r = − 0.33, r_s_ = 0.11, *p* < 0.001).

#### Responsiveness

##### Persistent injury

**Fig. 6:**
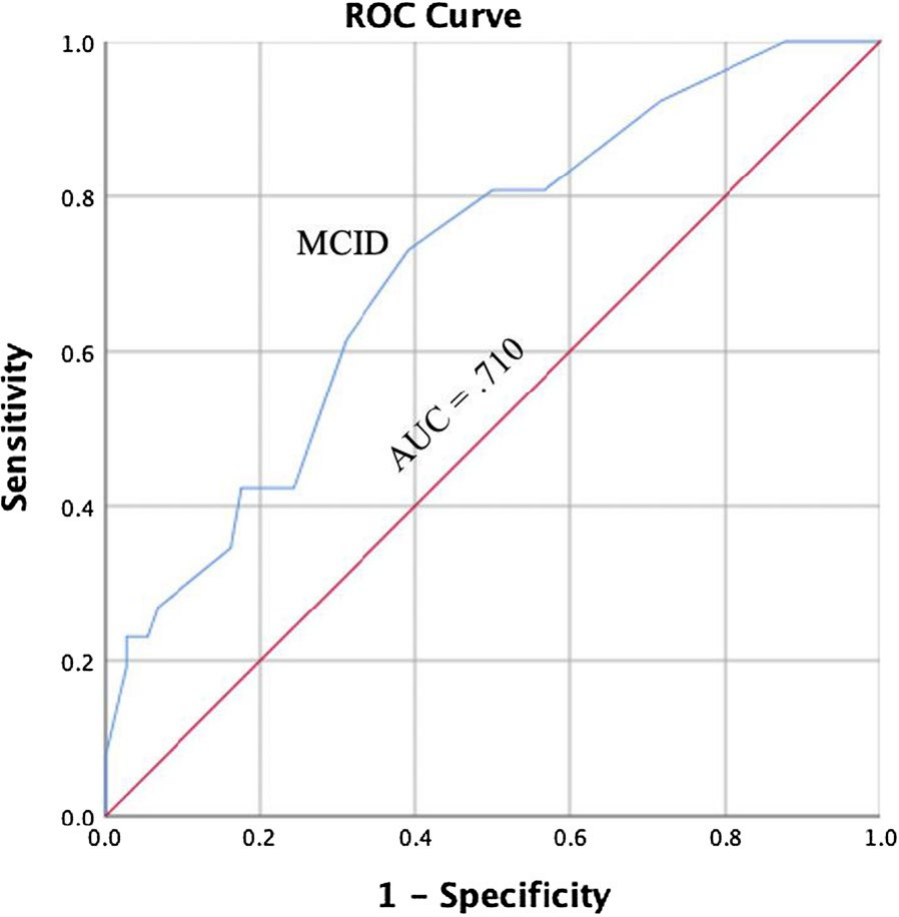
Receiver Operating Curve for Individuals with Persistent Injuries, Visit Two. AUC = area under the curve, MCID = minimal clinically important difference

One hundred individuals with a persistent injury, responded to the DPA SF-8 at visit one and two and were used for analysis. Of the 100 individuals, 26 reported experiencing a clinically significant change at the second visit. The AUC value for participants was 0.710 (95% confidence interval = 0.597, 0.822; *P* = 0.002; Fig. 6). The MCID value calculated for the ROC curve for visit 2 was 2.50 points (sensitivity = 0.731; 1 – specificity = 0.392).

**Fig. 7:**
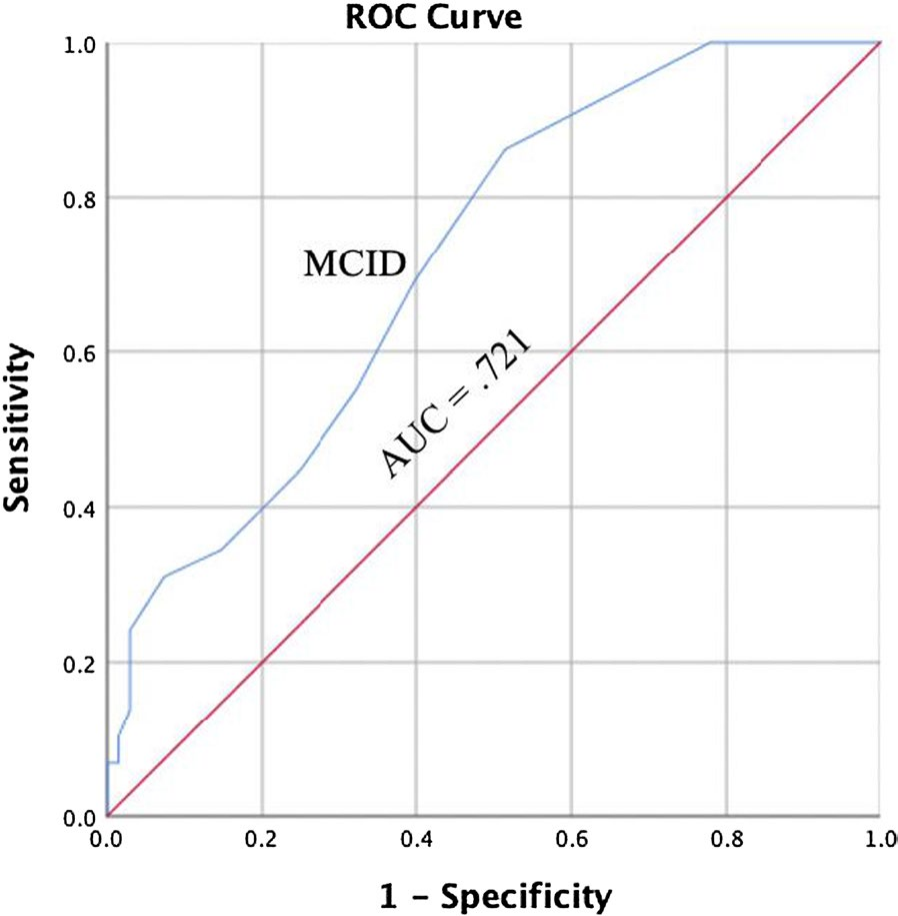
Receiver Operating Curve for Individuals with Persistent Injuries, Visit Three. AUC = area under the curve, MCID = minimal clinically important difference

Ninety-seven individuals with a persistent injury responded to the DPA SF-8 at visit 2 and 3 and were used for analysis. Of the 97 individuals, 29 reported experiencing a clinically significant change at visit three. The AUC value for participants was 0.721 (95% confidence interval = 0.616, 0.825; *P* = 0.001; Fig. 7). The MCID value calculated for the ROC curve for visit 3 was 1.50 points (sensitivity = 0.690; 1 – specificity = 0.397). The two values were averaged to create an MCID value of 2 points for individuals with persistent injuries.

##### Acute and subacute injuries

**Fig. 8:**
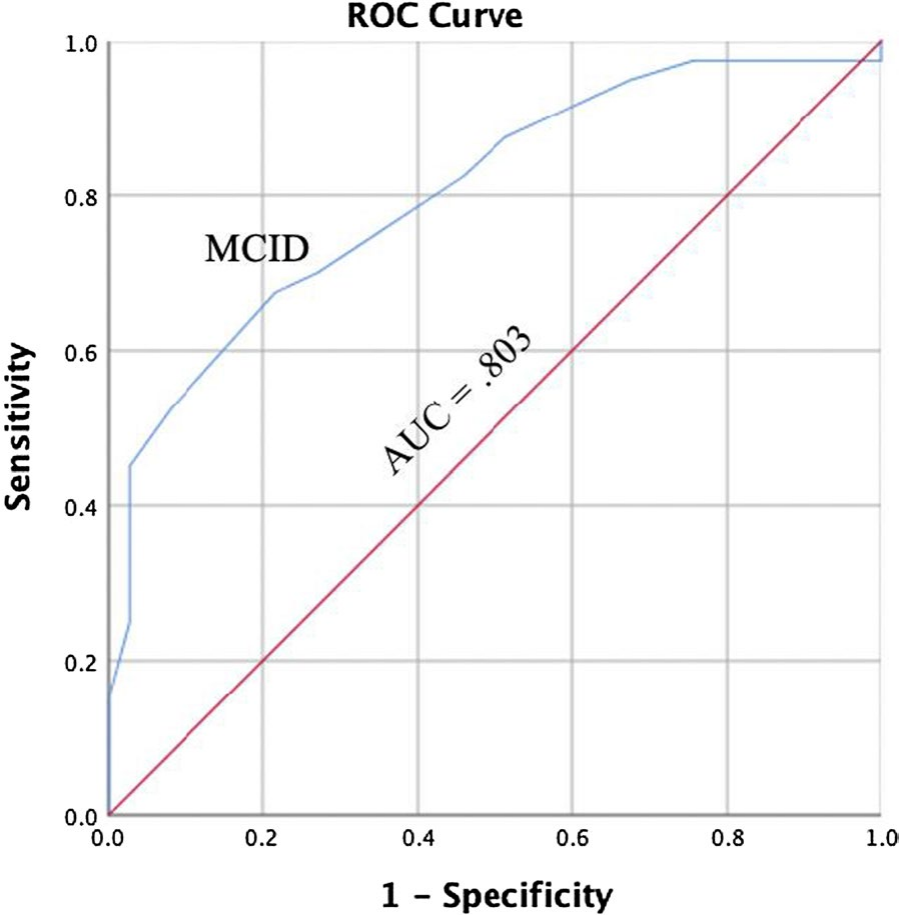
Receiver Operating Curve for Individuals with Acute and Subacute Injuries, Visit Two. AUC = area under the curve, MCID = minimal clinically important difference

Seventy-seven individuals with an acute or subacute injury, responded to the DPA SF-8 at visit 1 and 2 and were used for analysis. Of the 77 individuals, 40 reported experiencing a clinically significant change at visit two. The AUC value for participants was 0.803 (95% confidence interval = 0.706, 0.901; *P* < 0.001; Fig. 8). The MCID value calculated for the ROC curve on visit 2 was 3.5 point (sensitivity = 0.675; 1 – specificity = 0.216).

**Fig. 9:**
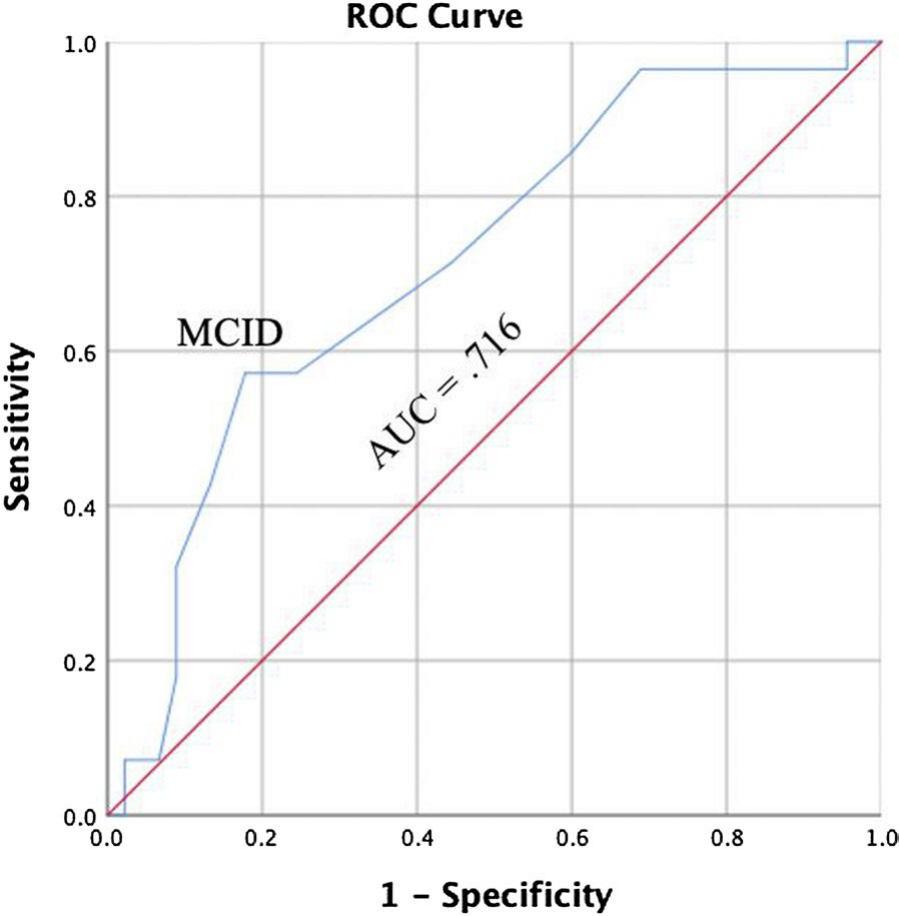
Receiver Operating Curve for Individuals with Acute and Subacute Injuries, Visit Three. AUC = area under the curve, MCID = minimal clinically important difference

Seventy-three individuals with an acute or subacute injury responded to the DPA SF-8 at visit 2 and 3 and were used for analysis. Of the 73 individuals, 28 reported experiencing a clinically significant change at visit 3. The AUC value for participants was 0.716 (95% confidence interval = 0.595, 0.837; *P* = 0.002; Fig. 9). The MCID value calculated for the ROC curve by visit 3 was 2.50 points (sensitivity = 0.571; 1 – specificity = 0.172). The two values were averaged to create an MCID value of 3 points for individuals with acute or subacute injuries.

### Invariance testing

#### Longitudinal (repeated assessment)

A total of 206 injured individuals responded to the DPA SF-8 at three time points (i.e., visits) and were used for analysis. The configural model (i.e., equal form) goodness of fit indices met recommended values (CFI = 0.981; χ^2^ [213] = 278.46; RMSEA = 0.039; Table 3). The metric model (i.e., equal loadings) passed both the CFI_DIFF_ test and the χ^2^_DIFF_ test, warranting examination of an equal latent variance model. The equal latent variance model passed both the CFI_DIFF_ and χ^2^_DIFF_ difference test, indicating variances were equal for first-order latent variables PHY and QOL across time.

**Table 3:** Goodness-of-fit indices for measurement invariance analyses across visit

	*χ*^*2*^	df	*χ*^*2*^_diff_ (df_diff_)	CFI	CFI_diff_	TLI	RMSEA
Visit 1	20.97	19	–	.997	–	.995	.023
Visit 2	30.40	19	–	.984	–	.976	.054
Visit 3	30.66	19	–	.988	–	.982	.055
Configural (equal form)	278.46	213	–	.981	–	.976	.039
Metric (equal loadings)	294.18	225	15.72(12)	.980	.001	.976	.039
Equal factor variances	310.43	229	31.97(16)	.977	.003	.972	.042
Scalar (equal indicator intercepts)	327.38	237	**48.92(24)**	.974	.006	.970	.043
Equal latent means	452.23	241	**173.77(28)**	.940	**.04**	.931	.065

CFI difference (CFIDIFF) above 0.01, and chi-square difference test (χ2DIFF) above a p-value cut-off of 0.01

The scalar model (i.e., equal indicator intercepts) slightly exceeded the χ^2^_DIFF_ test; however, it met the CFI_DIFF_ test, warranting examination of an equal latent means model. The equal latent means model did not pass either the CFI_DIFF_ or the χ^2^_DIFF_ test, indicating means for PHY and QOL were not equal across time. Analysis of means when not constrained to be equal indicated that individuals reported improved scores for PHY and QOL across time (i.e., repeated assessment).

#### Multigroup

##### Sex

A total of 452 individuals reported sex (male = 216; female = 236) at time one (i.e., visit one) and were used for analysis. The configural model (i.e., equal form) goodness of fit indices met recommended values (CFI = 0.983; χ^2^ [213] = 288.52; RMSEA = 0.039; Table 4). The metric model (i.e., equal loadings) passed both the CFI_DIFF_ test and the χ^2^_DIFF_ test, warranting examination of an equal latent variance model. The equal latent variance model passed both the CFI_DIFF_ and χ^2^_DIFF_ difference test, indicating variances were equal for first-order latent variables PHY and QOL across sex.

**Table 4:** Goodness-of-fit indices for measurement invariance analyses across sex

	*χ*^*2*^	df	*χ*^*2*^_diff_ (df_diff_)	CFI	CFI_diff_	TLI	RMSEA
Males (n = 216)	19.66	19	–	.999	–	.999	.013
Females (n = 236)	20.60	19	–	.998	–	.997	.019
Configural (equal form)	40.26	38	–	.999	–	.998	.011
Metric (equal loadings)	51.86	44	11.60(6)	.995	.004	.994	.020
Equal factor variances	52.16	46	11.90(8)	.996	.003	.996	.017
Scalar (equal indicator intercepts)	59.98	50	19.72(12)	.994	.005	.993	.021
Equal latent means	65.27	52	25.01(14)	.992	.007	.991	.024

The scalar model (i.e., equal indicator intercepts) passed both the CFI_DIFF_ test and the χ^2^_DIFF_ tests, warranting examination of an equal latent means model. The equal latent means model passed both the CFI_DIFF_ and the χ^2^_DIFF_ test, indicating means for PHY and QOL were equal across sex.

##### Activity level

A total of 392 injured individuals reported their activity level (low = 105, medium = 179, high = 133) at time one (i.e., visit one) and were used for analysis. The configural model (i.e., equal form) goodness of fit indices met recommended values (CFI = 0.995; χ^2^ [57] = 60.58; RMSEA = 0.013; Table 5). The metric model (i.e., equal loadings) passed both the CFI_DIFF_ test and the χ^2^_DIFF_ test, warranting examination of an equal latent variance model. The equal latent variance model passed both the CFI_DIFF_ and χ^2^_DIFF_ difference test, indicating variances were equal for first-order latent variables PHY and QOL across activity level. The scalar model (i.e., equal indicator intercepts) passed both the CFI_DIFF_ test and the χ^2^_DIFF_ tests, warranting examination of an equal latent means model. The equal latent means model passed both the χ^2^_DIFF_ test and the CFI_DIFF_ difference test, indicating means were equal for first-order latent variables PHY and QOL across activity levels.

**Table 5:** Goodness-of-fit indices for measurement invariance analyses across activity level

	*χ*^*2*^	df	*χ*^*2*^_diff_ (df_diff_)	CFI	CFI_diff_	TLI	RMSEA
Low (n = 102)	21.04	19	–	.995	–	.992	.033
Medium (n = 169)	19.01	19	–	1.00	–	1.00	.002
High (n = 121)	20.51	19	–	.996	–	.993	.026
Configural (equal form)	60.58	57	–	.997	–	.996	.013
Metric (equal loadings)	76.75	69	16.17(12)	.994	.003	.992	.017
Equal factor variances	87.24	73	26.66(16)	.988	.009	.986	.022
Scalar (equal ndicatorrcepts)	85.93	81	25.35(24)	.996	.001	.996	.013
Equal latent means	95.91	85	35.33(28)	.991	.006	.991	.018

##### Injury category

A total of 329 individuals reported having a persistent (n = 177) or a subacute or acute injury (n = 161) at time one (i.e., visit one) and were used for analysis. The configural model (i.e., equal form) goodness of fit indices met recommended values (CFI = 1.0; χ^2^ [38]  = 30.89; RMSEA < 0.001; Table 6). The metric model (i.e., equal loadings) passed both the CFI_DIFF_ test and the χ^2^_DIFF_ test, warranting examination of an equal latent variance model. The equal latent variance model passed both the CFI_DIFF_ and χ^2^_DIFF_ difference test, indicating variances were equal for first-order latent variables PHY and QOL across injury category.

**Table 6:** Goodness-of-fit indices for measurement invariance analyses across injury category

	*χ*^*2*^	df	*χ*^*2*^_diff_ (df_diff_)	CFI	CFI_diff_	TLI	RMSEA
Persistent (n = 177)	10.94	19	–	1.00	–	1.00	.000
Acute/subacute (n = 161)	18.79	19	–	1.00	–	1.00	.000
Configural (equal form)	30.89	38	–	1.00	–	1.00	.000
Metric (equal loadings)	40.08	44	9.19(6)	1.00	NC	1.00	.000
Equal factor variances	40.88	46	9.99(8)	1.00	NC	1.00	.000
Scalar (equal indicator intercepts)	43.47	50	12.58(12)	1.00	NC	1.00	.000
Equal latent means	55.22	52	24.33(14)	.997	.003	.997	.014

The scalar model (i.e., equal indicator intercepts) passed both the CFI_DIFF_ test and the χ^2^_DIFF_ tests, warranting examination of an equal latent means model. The equal latent means model passed both the CFI_DIFF_ and χ^2^_DIFF_ difference test, indicating means were equal for first-order latent variables PHY and QOL across injury category.

## Discussion

Patient-centered care and EBP are core competencies for health care professionals. The importance of EBP has led to an increase in research involving clinical outcomes (e.g., physiological findings, patient self-reported instruments); current recommendations emphasize collecting patient focused measures (e.g., the patient’s perspective and experience of their range of motion) [7]; thus increasing the need for psychometrically sound patient reported outcome measures (PROMs) of health [3]. Disablement has been proposed as a valuable multi-dimensional construct for patient care; however, selecting an appropriate disablement PROM to assess disablement may depend on the specific subgroups of patients completing the scale [11].

The DPA SF-8 [13], assesses two factors of disablement: PHY and QOL component [12, 13]. The DPA SF-8 exceeded contemporary standards for model fit and [12, 13]; however, further analysis was necessary to ensure the DPA SF-8 was psychometrically sound and could accurately assess disablement across subgroups and time [15, 16]. Therefore, the purposes of our study were to establish the DPA SF-8 scale reliability, validity, sensitivity to change, responsiveness, and longitudinal and multi-group invariance.

### DPA SF-8 scale structure

The CFAs at all three visits exceeded recommended model fit indices, thus confirming the scale structure of the DPA SF-8 [12, 13]. This study, however, was the first to use a large heterogeneous sample of adolescents and adults who responded to the 8-item scale. The total scores on the DPA SF-8 by injury classification (Table 2) were similar to scores reported in previous research [13]. Individuals with a persistent or acute injury reported higher overall scores (i.e., more disablement and lower quality of life) than healthy individuals who reported lower overall scores (i.e., less disablement and higher quality of life). The correlation values between the first-order latent variables PHY and QOL (r = 0.40–49, r_s_ = 0.16–24) across visits were also similar to previous research [12, 13]; the findings support that the PHY and QOL constructs of disablement are unique constructs [13].

Overall, the scale structure findings indicate exceptional model fit for the DPA SF-8 in respondents who only answer the 8-items, and suggest the DPA SF-8 continues to resolve item redundancy and multicollinearity issues found in the DPA Scale or DPA SF-10 [11–13]. Although the scale was designed for use in the physically active, our full sample included a small percentage (n = 25, 5.2%) of individuals with extremely low levels of physical activity (i.e., activities of daily living). The excellent model fit with those individuals included, as well as the excellent model fit in studies excluding extremely low levels of physical activity individuals [12, 13], implies the scale may be psychometrically sound in both groups. However, future research should assess the scale structure of the DPA SF-8 in a larger group of individuals with extremely low levels of physical activity, as well as in inactive individuals. Additionally, multi-group invariance between physically active and inactive individuals should be performed to ensure scale structure is supported across these groups.

### Reliability of the DPA SF-8

Cronbach’s alpha for PHY and QOL were within recommend values, which support sound internal consistency of the constructs and reduced risk of multicollinearity in the scale. The ICC value (0.924) calculated across three time points (i.e., initial visit, visit 2 = 3–5 days post initial visit, visit 3 = 7–10 days post-initial visit) indicated excellent scale reliability [32]. Our results were similar to the original ICC value (0.943) found for the 16-item DPA Scale in injured individual across two time points, 24 h apart [11]; our ICC value was higher than the reliability value (0.792) reported in soccer players tested on the 16-item scale during preseason, one week apart [42]. Our results indicate a true change in a patient’s overall disablement (i.e., total DPA DF-8 score) when completing the DPA SF-8 multiple times is likely less than 6 points (MDC = 5.83; an 18.2% change on the 0–32 scale), which was improved from a previously reported MDC value of 12.48 (a 19.5% change on the 0–64 scale) for the DPA Scale [42]. The improved internal consistency and MDC values of the DPA SF-8 were expected; the DPA SF-8 has improved precision and model fit, as well as fewer items and decreased item redudancy, compared to the original 16-item DPA Scale [12, 13].

### Criterion (concurrent) validity

Criterion validity was assessed by examining the correlations between the DPA SF-8 and the scores on the GF scale, NPRS, and PSFS. The significant inverse relationship between the GF scale and DPA SF-8 is consistent with previous findings [11]; however, the second-order latent variable correlation values across all participants and timepoints (i.e., visits) in our study were lower (r = − 0.63, r_s_ = 0.40 [visit 1], r = − 0.56, r_s_ = 0.31 [visit 2], and r = − 0.65, r_s_ = 0.42 [visit 3]) than previous findings (r = − 0.714, r_s_ = 0.51 for persistent and r = − 0.751, r_s_ = 0.56 for acute injuries) for the original 16-item scale [11]. The small difference in correlational values may be the result of study or scale differences. We utilized a larger and more diverse participant pool with a higher mean age than the previous study [11] and we included a small portion of healthy individuals; it is possible that differences in participant age between the studies or the healthy participants included in our analysis resulted in slightly different responses across items or scales. For example, people who are healthy should not be processing change from injury, while people of different ages who are injured process changes across health dimensions (e.g., physical function, quality of life) differently across the lifespan [43]; those differences may have altered the correlational values between the scales. Another potential explanation is reduced item redundancy in the DPA SF-8 due to the decreased number of PHY questions (i.e., 4 items compared to 12) present in the short form compared to the DPA Scale; the removal of highly correlated items assessing physical functioning may have also reduced the correlation between the GF scale and the DPA SF-8.

The assessment of concurrent validity should also involve correlating the DPA SF-8 to other relevant scales because the DPA SF-8 is a multidimensional scale that allows summative (i.e., scale total) and construct (i.e., PHY and QOL) scoring. The correlational directions (e.g., inverse with GF scale and PSFS) and magnitudes met expectations and support concurrent validity. The second-order latent variable correlational analysis indicated significant positive relationships between the DPA SF-8 and the NPRS across visits, with an inverse significant relationship between the DPA SF-8 and the PSFS across visits. The first order latent variable correlations between the PHY construct of the DPA SF-8 and the NPRS and PSFS demonstrated a similar pattern of direction and magnitude across visits. The first order correlations were also significant between QOL construct of the DPA SF-8 and the NPRS and PSFS; however, correlation values between these scales and the QOL construct were, as expected, lower than those found with the PHY construct.

The overall correlational findings support the validity of the DPA SF-8. The DPA SF-8, like the DPA Scale, was significantly and appropriately correlated with the GF Scale providing some evidence of criterion validity. Additionally, the DPA SF-8 PHY construct was highly correlated with related unidimensional scales (i.e., NPRS and PSFS) designed to measure components found in that dimension. The DPA SF-8 QOL construct was correlated with related unidimensional scales (i.e., GF Scale, NPRS, and PSFS); the correlation values fit proposed theory in that the correlations were in the same direction but of lower magnitude to those found with the PHY construct. Further, the correlation values between the DPA SF-8 PHY and QOL constructs and the GF Scale, NPRS, and PSFS increased over visits which indicated that patient improvement was being identified across both SF-8 constructs and the other scales in a more similar pattern. Finally, the primarily adult population in our study more strongly defined (i.e., more heavily weighted) improvement through physical health changes, as opposed to QOL changes, which is consistent with the expectations developed in previous research [43]. Future research should be completed to further establish the validity of the QOL subscale by correlating the construct to another previously established quality of life instrument.

### Receiver operating curve responsiveness

We also evaluated the ability of the DPA SF-8 to detect change over time, or the responsiveness of the scale [34, 35], using a ROC curve analysis. Previous research utilized the GRoC to classify individuals into either a clinically significant group or a stable group to develop MCIDs [11, 25]. We chose to utilize three additional outcome measures (i.e., NPRS, PSFS, GF) in addition to the GRoC for classification into the grouping for two reasons: (1) the GRoC has been scrutinized for poor reliability and recall bias [36], and (2) the multidimensional nature of the DPA SF-8 was better represented by utilizing multiple instruments that represented the depth of the unique constructs/items of the DPA SF-8.

The four ROC curves were then constructed based on our groupings at two time points (i.e., visit two and visit three): two for individuals with persistent injuries and two for individuals with acute or subacute injuries. The four AUC values (range = 0.710–0.803) for the ROC curves were statistically significant and within the moderately high range, indicating the scale could detect meaningful change from the patient perspective. Overall, our range of AUC values was slightly narrower (i.e., top end was lower) than was found for the DPA Scale (range = 0.702–0.911); however, our sample was significantly larger and more diverse, and did not have a group (i.e., acute) where all members experienced a significant change [11].

We calculated the MCID values using the ROC curve for two groups of respondents: (1) individuals with a persistent injury, (2) and individuals with acute and subacute injuries. The MCID values are beneficial for providing clinicians and researchers with insight into true clinical change as perceived by the patient. Our results indicate patients with a persistent injury will have likely experienced a clinically significant change with a 2 point or greater change (6% or greater change) in the total DPA SF-8 score. For those with a subacute or acute injury, a clinically significant change will likely have occurred if a 3 point or greater (9.4% or greater change) change is reported on the total DPA SF-8 score. The MCID values for the DPA SF-8 are lower than those reported for the DPA Scale for persistent (6 points; 9.4%) and acute (9 points; 14.1%) injuries [11]; however, the lower values are expected given the removal of items (16 to 8) resulting in improved model fit, reduced item redundancy, resolved multicollinearity, and improve scale precision.

Our findings, however, may be limited by our sample and methodology. The established MCID values may have improved accuracy if group classification included a component to more effectively assess and classify change in the QOL construct. For example, adolescents weigh responses more heavily to mental health changes than adults [43], and those changes may not be effectively captured in any of the methodologies utilized to establish MCIDs for the DPA Scale or the DPA SF-8. Thus, future research may be needed to establish MCID for the sub-constructs of the scales, across different age groups, or using methods which classify change more effectively across both physical and mental health dimensions to better represent the multi-factorial nature of the DPA Scale and DPA SF-8.

### Multi-group and longitudinal invariance

Our study is the first to assess invariance of the DPA SF-8 across time visits and groups of interest (i.e., sex, injury classification, activity level). Invariance testing is necessary to ensure the association between the PHY and QOL latent variables, and their items, are stable and relatively equal over visits and between groups [15, 29, 44]. An instrument that is invariant allows for comparisons across group and time (i.e., visit) by confirming individuals are interpreting the items and meaning of the items similarly, which provides evidence that score changes or group differences are true changes/differences as opposed to differences due to other group/time attributes or measurement error [15, 29].

Our results indicate the DPA SF-8 was invariant across all our analyses, which allows clinicians and researchers to use the scale to compare differences in the tested groups (e.g., sex, physical activity level) or to assess individual changes in scores over the treatment period. We did not find group mean differences in the PHY or QOL constructs across sex or physical activity level. Our results differ from previous research where individuals who engaged in physical activity report higher scores on quality of life [45]; however, our results may have been confounded by the physical activity group including participants who were currently suffering an injury which likely would have reduced QOL scores compared to those who were uninjured but physically active.

Our invariance results also support the validity and responsiveness of the DPA SF-8. The DPA SF-8 was invariant across the persistent and acute/sub-acute injury groups which indicates the scale may be used to assess differences in disablement across the two groups. Further, the responses of the injured participants were invariant across visits and revealed significant improvements in their health status (i.e., reduced physical impairment and disability and improved quality of life) across repeated measures. The DPA SF-8 revealing significant improvement over visits for those suffering injuries would be expected when natural healing and care from their healthcare provider is occurring across the repeated measures.

Unfortunately, our sample did not include a large enough number of healthy participants to include this group in the multi-group invariance test procedures with the persistent and acute/sub-acute injury groups. Researchers have indicated the DPA Scale did not demonstrate multi-group invariance across injured and uninjured participants [13]; further research is needed to establish if the DPA SF-8 is invariant between these groups, ensuring item-level bias does not occur due to group attributes. Clinically, establishing invariance across injured and uninured participants helps ensure item interpretation and measurement are measured consistently across these two groups, which is valuable when the DPA SF-8 is used to inform return to play or discharge from care decisions (i.e., when patients transition from injured to healthy).

### Clinical implications

Healthcare professional working with patients who participate in sport or exercise and suffer a musculoskeletal injury need a valid instrument to assess patient perceptions of the injury and the perceived effectiveness of care provided for that injury. Our study results indicate the DPA SF-8 is a reliable, valid, and responsive multi-dimensional instrument that can be used with those who suffer a musculoskeletal injury during physical activity (i.e., sport or exercise). Clinicians and researchers may use the DPA SF-8 to assess treatment efficacy across repeated measures or to compare scores between certain groups; however, caution is warranted if scores are being compared across injured and uninjured respondents at this time. The MDC (5.83 points) and MCID (acute/subacute = 3; persistent = 2) values support the responsiveness of the scale: 1) clinicians and researchers may interpret a real change outside of measurement error has occurred when a change greater or equal to 6 points has occurred; 2) a clinically significant change important to the patient can be interpreted when a 2 point or greater or 3 point or greater change is reported by those with a persistent or acute/subacute injury, respectively. Our results also confirm previous findings [13] that the PHY and QOL constructs are unique dimensions captured within the scale to measure health status in the physically active [46].

Our results support previous findings [12, 13] for scoring the individual constructs (i.e., PHY and QOL); however, our study is the first to establish MDC values the for the PHY (MDC = 4) and QOL (MDC = 5) constructs. Clinicians may use the MDC values to determine when a patient reports a change in each construct that is greater than the expected error for repeated completion of each construct; however, further research is needed to establish MCIDs for each construct. While examining the individual construct scores is likely a more accurate portrayal of health status [14], cumulative scores can be created and assessed (e.g., MDC values, MCID values) to provide clinicians insight into the overall health status of their patient. Clinicians should consider whether the improvements in DPA SF-8 cumulative scores are primarily driven by changes in physical function assessed by the items in the PHY and QOL constructs as opposed to true changes in mental health (i.e., QOL). Clinicians who use cumulative scores should also assess subdimension scores and consider the use of additional wellness or mental health status questionnaires when appropriate for a specific patient case.

### Limitations and future research

Although our study used a reasonably large heterogenous population, most of our respondents (mean age = 28 years) were either in the emerging and early adulthood stages of human development. Our cross-sectional sample had smaller participation from members in other stages (e.g., adolescents, middle adulthood, late adulthood) of human development. Therefore, future research should establish model fit and multi-group and longitudinal invariance of the DPA SF-8 across these age groups as appropriate for various clinical settings. Our sample also included a small percentage of individuals who had extremely low levels of physical activity; however, the group of individuals was too small to include in multi-group invariance testing. Future research should be conducted using a larger sample of extremely low physical active individuals and inactive individuals to ensure scale structure is sound in these groups, while also performing multi-group invariance testing (i.e., active vs. inactive individuals) to ensure group differences are not due to measurement error. Similarly, the MDC and MCID values may be different across groups (e.g., adolescent, emerging adult groups, low activity, high activity) and future research should seek to determine if those values vary across relevant clinical groups.

We used a similar protocol as previous research [11] to establish group classifications for clinically significant improvement; however, this exact method has not been replicated in the literature and the methods utilized may not best capture change across a multidimensional instrument. Therefore, future research should assess the responsiveness of the scale in a diverse sample of individuals with different instruments that adequately capture the depth and uniqueness of the PHY and QOL constructs of the DPA SF-8 to improve accuracy of classifications and MCID values. Additionally, we used previously established methods [11] for assessing construct validity by correlating the GF scale to the DPA SF-8; however, we also conducted second and first order correlations between the DPA SF-8 and the GF scale, NPRS, and PSFS to assess construct validity. While the results support the validity of the DPA SF-8, further research is warranted to establish the validity of the QOL construct, as well as validity of a cumulative DPA SF-8 score as a measure for health status. Finally, while we had a sufficient sample size for much of our analysis work, we were limited by sample size in certain cases (e.g., multi-group invariance testing including a healthy group for comparison to injured participants); we also experienced participant dropout over the course of the study (i.e., participants being unable to return for 2nd or 3rd visits due to COVID-19). Thus, future research using large samples with higher completion rates for all three time points (i.e., visits) would be valuable to confirm or refute certain study findings (e.g., MCID values).

### Conclusions

The DPA SF-8 met the strictest CFA recommendations without the need for scale modification in respondents who only answered the 8-items included in the scale. The DPA SF-8 also met all criteria for applied multi-group and longitudinal invariance tests, which indicates the scale may be used to assess for differences between the groups or across time. Our overall analysis indicates the DPA SF-8 is a valid, reliable, and responsive instrument to assess patient improvement in the physically active population.

## Supplementary Information

**Additional file 1: Table S1**. Study definitions and termnology.

## Data Availability

Data can be made available upon reasonable request to corresponding author.

## Abbreviations

EBP: Evidence-based practice
PROM: Patient reported outcome measure
DPA: Disablement in Physically Active
DPA SF-8: Disablement in Physically Active Scale Short Form-8
MCID: Minimal clinically important difference
CFA: Confirmatory factor analysis
GRoC: Global Rate of Change Scale
PHY: Physical
QOL: Quality of life
NPRS: Numeric Pain Rating Scale
GF: Global Functioning
PSFS: Patient Specific Functional Scale
AMOS: Analysis of Moment Structures
CFI: Comparative Fit Index
TLI: Tucker-Lewis Index
RMSEA: Root Mean Square Error of Approximation
IFI: Bollen's Incremental Fit Index
CMIN: Likelihood ratio statistic
ICC: Intraclass correlation coefficients
SEM: Standard error of measurement
MDC: Minimal detectable change
ROC: Receiver Operating Characteristics
AUC: Area under the curve
CFI_DIFF_: CFI difference
χ^2^_DIFF_: Chi-square difference test
r: Correlation coefficient
r_s_: Square of correlation coefficient
