# Supplementary material for: Disablement in the Physically Active Scale Short Form-8: psychometric evaluation
Source: BMC Sports Sci Med Rehabil. 2021 Dec 14;13:153. doi: 10.1186/s13102-021-00380-3 (PMC8669416; doi:10.1186/s13102-021-00380-3)
Supplement: Supplementary file 1 — Additional file 1: Table S1. Study definitions and termnology. [file 13102_2021_380_MOESM1_ESM.docx]

Supplemental Material

**Supplemental Table 1. Study Definitions and Terminology**

| **Terminology** | **Definition** |
| --- | --- |
| Physically Active | “An individual who engages in athletic, recreational, or occupational activities that require physical skills and who uses strength, power, endurance, speed, flexibility, range of motion, or agility at least 3 days per week.” ^11^ |
| **Injury Classification** ^11^ | |
| Healthy | “Free from musculoskeletal injury and fully able to participate in sport or activity.” |
| Acute Injury | “A musculoskeletal injury that precludes full participation in sport or activity for at least 2 consecutive days (0-72 hours post-injury).” |
| Subacute Injury | “A musculoskeletal injury that precludes full participation in sport or activity for at least 2 consecutive days (3 days to 1-month post-injury).” |
| Persistent Injury | “A musculoskeletal injury that has been symptomatic for at least 1 month.” |
| Chronic Injury | “Pain that consistently does not get any better with routine treatment or non-narcotic medication.” |
| **Activity Level Classification** ^47^ | |
| Extremely low | “No activity beyond baseline activity (baseline activity refers to ‘light-intensity activities [e.g., standing, walking, lifting weighted objects] of daily life).” |
| Low | “Activity beyond baseline, but fewer than 150 minutes of moderate intensity exercise per week (moderate activity includes activities such as brisk walking, yoga, lifting weights, etc.).” |
| Medium | “150-300 minutes of moderate intensity activity per week”. |
| High | “More than 300 minutes of moderate intensity activity per week.” |
| **Athlete Status** ^11^ | |
| Competitive | “A participant who engages in a sport activity that requires at least 1 pre-participation examination, regular attendance at scheduled practices and/or conditioning sessions and a coach who leads practices and/or competitions.” |
| Recreational | “Participants who meet the criteria for physical activity and participate in sport, but do not meet the criteria for competitive status.” |
| Occupational | “Participants who meet the criteria for physical activity in occupation or recreation, but do not meet the criteria for competitive or recreational athlete.” |
| Physically Active in Activities of Daily Living | “Participants who do not meet the criteria for any “athlete” category, but who are physically active through their daily activities (e.g., physically active for at least 30 minutes per day, 3 days per week).” |
